# Forças Anteriores Proeminentes do QRS Durante Suboclusão Transitória do Tronco da Coronária Esquerda

**DOI:** 10.36660/abc.20180363

**Published:** 2020-09-11

**Authors:** Andrés Ricardo Pérez-Riera, Raimundo Barbosa-Barros, Rodrigo Daminello Raimundo, Luiz Carlos de Abreu, Marcos Célio de Almeida, Kjell Nikus

**Affiliations:** 1 Centro Universitario Saúde ABC Santo AndréSP Brasil Centro Universitario Saúde ABC,Santo André, SP - Brasil; 2 Hospital de Messejana Dr. Carlos Alberto Studart Gomes FortalezaCE Brasil Hospital de Messejana Dr. Carlos Alberto Studart Gomes,Fortaleza, CE - Brasil; 3 Universidade de Brasília Instituto de Biologia-Genética e Morfologia BrasiliaDF Brasil Universidade de Brasília - Instituto de Biologia-Genética e Morfologia,Brasilia, DF - Brasil; 4 Heart Center Tampere University Hospital Faculty of Medicine and Life Sciences Tampere Finlândia Heart Center, Tampere University Hospital and Faculty of Medicine and Life Sciences,Tampere – Finlândia

**Keywords:** Oclusão Coronária, Tronco Arterial, Síndrome Coronária Aguda, Fibrinolíticos, Intervenção Coronária Percutânea, Angina Estável, Eletrocardiografia/métodos

## Introdução

O tronco de coronária esquerda (TCE) origina-se do seio esquerdo de Valsalva, passa entre a artéria pulmonar principal e o apêndice atrial esquerdo antes de entrar no sulco coronário e bifurca-se na artéria descendente anterior esquerda (DAE) e na artéria circunflexa esquerda (CXE). Na maioria dos indivíduos, o TCE supre ≈75% do ventrículo esquerdo (VE).^1^ Uma estenose significativa, que pode causar angina estável e/ou síndrome coronariana aguda, coloca o paciente em risco de insuficiência ventricular esquerda aguda fatal e arritmias malignas. O prognóstico do paciente com doença de TCE pode ser melhorado com a revascularização miocárdica (RM). Com aprimoramento técnico e medicação antitrombótica eficaz, a intervenção coronária percutânea (ICP) evoluiu como uma modalidade terapêutica alternativa. Em pacientes com doença de TCE grave com complexidade anatômica baixa a intermediária, tanto a RM quanto a ICP são métodos eficazes de revascularização com taxas comparáveis de morte, infarto do miocárdio e acidente vascular cerebral em longo prazo.^2^ Os pacientes mais adequados para implante de stent no TCE são aqueles com doença ostial isolada/doença de TCE média, doença de TCE protegido e aqueles submetidos a implante de stent eletivo. Em um estudo recente, foram relatados 8% de mortalidade e 8% de taxa de revascularização da lesão-alvo durante o seguimento de um ano.^3^

## Descrição do caso

**Figura 1:**
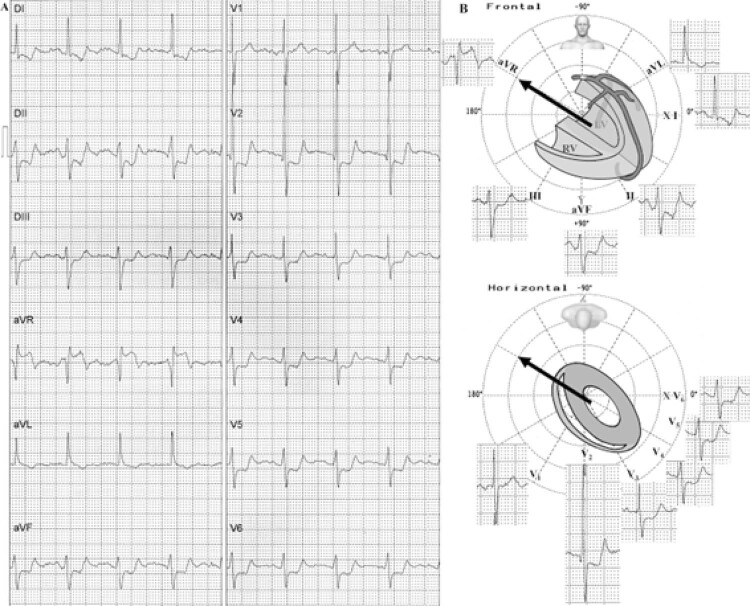
ECG na admissão (A) e o vetor de lesão nos planos frontal e horizontal (B). A) Infradesnivelamento generalizado do segmento ST em I, II, III (II> III) e VF e das ondas V2 a V6. Infradesnivelamento difuso do segmento ST nas derivações ínfero-laterais (≥7 derivações com Infradesnivelamento do segmento ST) e supradesnivelamento recíproco do segmento ST na derivação aVR. Além disso, bloqueio fascicular anterior esquerdo (BFAE) atípico, eixo QRS -40°, SIII >SII e ausência de onda q inicial em I e aVL pela ausência do primeiro vetor do septo médio esquerdo (no BFAE típico, os primeiros vetores de 10-20 ms são direcionados para +120°).4 B) Plano frontal (PF): o vetor de lesão ST (seta) é direcionado para cima e para a direita, apontando para a derivação aVR (-150°). Quando esse vetor está localizado entre -90 ° e ± 180° no PF, é indicativo de obstrução do TCE em até 100% dos casos5, infradesnivelamento do segmento ST nas derivações inferiores com STII >STIII; Plano horizontal: o vetor de lesão de ST é direcionado para a direita e esquerda (seta), perpendicular a V1. Infradesnivelamento do segmento ST de V2 a V6.

**Figura 2:**
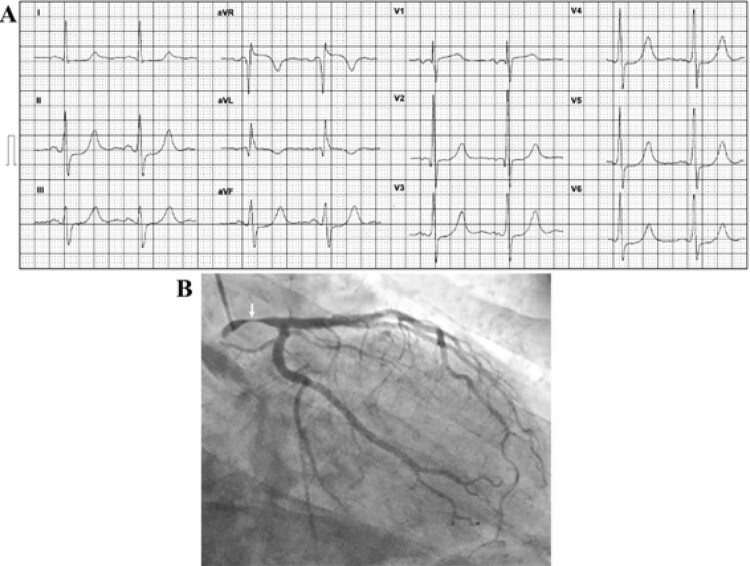
– A) ECG realizado 30 dias antes: aumento atrial esquerdo, forças anteriores proeminentes do QRS em V2 com padrão de qRs em V1-V2, voltagem da onda R em V2 >15 mm (23 mm), tempo de pico da onda R prolongado nas derivações precordiais direitas (≥35 ms), supradesnivelamento do segmento ST em aVR (≥1 mm), infradesnivelamento mínimo do segmento ST nas derivações inferiores e de V3 a V6; essas alterações discretas podem levantar a suspeita de doença de TCE e algum grau de LSFB. Nota: este ECG foi considerado “normal” pelo clínico!! B) Angiografia coronariana na projeção oblíqua craniana anterior direita: nesta projeção, observa-se uma suboclusão crítica do TCE (seta) na porção média.

Um homem caucasiano de 72 anos de idade veio ao pronto-socorro com queixa de dor torácica opressiva prolongada em repouso há uma hora associada a diaforese fria e dificuldade respiratória. Ele tinha histórico de diabetes mellitus tipo 2 e dislipidemia, detectada quatro anos antes. Dois meses antes, ele tinha apresentado dor precordial opressiva a esforços moderados, que desaparecia rapidamente com repouso. A Figura 1 mostra o ECG na admissão e a Figura 2-A um ECG realizado 30 dias antes. A angiografia coronariana mostrou suboclusão (estenose de 91 a 99% de diâmetro) na porção média do TCE ( Figura 2-B ). A RM foi proposta imediatamente, mas o paciente recusou. Ele foi submetido à ICP com implante de stent farmacológico (SF) com sucesso, sem complicações intra-hospitalares. Durante os 6 meses de seguimento, não foi necessária revascularização da lesão-alvo no TCE. O paciente permaneceu assintomático mesmo com esforço físico e os vários ECGs de seguimento foram normais.

## Discussão

Um eletrocardiograma realizado devido a sintomas de angina estável 30 dias antes da internação hospitalar mostrou um padrão sugestivo de doença de TCE e possivelmente algum grau de suboclusão transitória do tronco da coronária esquerda (LSFB, do inglês *transient left septal fascicular block* ).^4 , 5^ Esses “achados mínimos” no cenário de angina estável devem alertar o médico sobre a possibilidade de isquemia miocárdica grave em pacientes sem uma explicação lógica para os achados do ECG, como hipertrofia ventricular esquerda com *strain* na cardiopatia estrutural. Ambas as características do ECG são evidentes, com achados isquêmicos mais pronunciados no ECG realizados na admissão quando o paciente apresentava síndrome coronariana aguda.

Vários manuscritos sucessivos do nosso grupo e de outros mostraram que uma grande proporção de casos com LSFB, manifestado por forças anteriores proeminentes do QRS, é causada por obstrução proximal crítica da DAE antes de seu primeiro ramo perfurante septal.^6 - 9^ Como a DAE é uma continuação do TCE, uma obstrução significativa do TCE pode levar à isquemia na porção média e no território apical do ventrículo esquerdo, onde fica o fascículo septal esquerdo, causando a LSFB.

Na presença de LSFB, a sequência de ativação ventricular começa apenas em dois pontos:

- A base do músculo papilar anterolateral (MPA) da válvula mitral dependente do fascículo anterior esquerdo (FAE) na parede parasseptal anterior, logo abaixo da inserção do MPA (vetor 1_AM_);
- A base do músculo papilar posteromedial (MPP) da válvula mitral dependente do fascículo posterior esquerdo (FPE). Está localizado na parede parasseptal posterior, a cerca de um terço da distância do ápice até a base (vetor posteroinferior - 1_PI_). Esses dois vetores iniciais têm direções opostas e se cancelam com predominância mínima do vetor 1_PI_ direcionado para trás ( Figura 3 ). Isso explica a ausência da convexidade inicial normal à direita do *loop* QRS (despolarização ventricular) no plano horizontal, dependente do vetor septal 1_AM_ (ou vetor Penaloza-Tranchesi).^10^ Em seguida, o estímulo é direcionado para a região médio-septal ou parasseptal esquerda, bloqueada por inúmeras áreas do sistema de Purkinje, deslocando as forças para a frente e para a esquerda, resultando em forças anteriores proeminentes **(** FAP). A Figura 4 mostra dois casos em que a anatomia trifascicular do ramo esquerdo do feixe de His é evidente. Ironicamente, ambos os casos vêm da escola de eletrocardiografia que cunhou o conceito bifascicular do ramo esquerdo do feixe de His.^11^
  **Figura 3:**
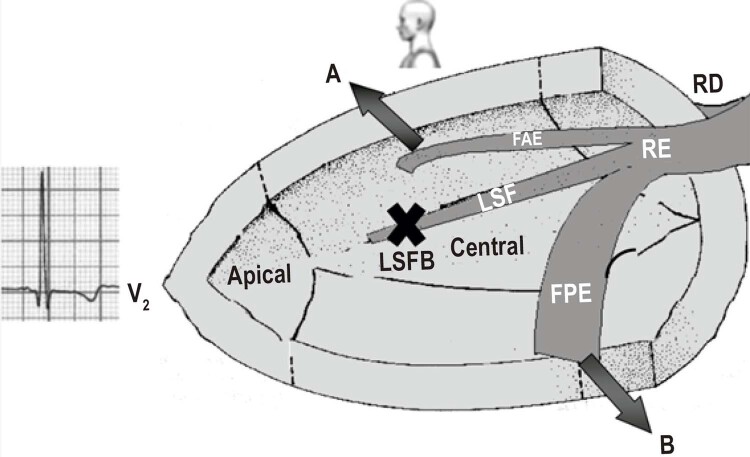
– Esboço mostrando a ativação ventricular inicial nos casos de LSFB. Ramo esquerdo do feixe de His com suas três divisões, em uma projeção sagital esquerda. O FAE termina na base do MPP da válvula mitral. O FPE termina na base do MPP da válvula mitral. Como os vetores de ativação dependem dos fascículos anterossuperior (A) e posteroinferior (B) vão em direções opostas, eles se cancelam, com predominância mínima do FPE. Esse fenômeno explica a frequente onda q inicial nas derivações precordiais direitas na presença de BFPE. Observe a ausência do primeiro vetor 1AM, dependente do LSF. RE: ramo esquerdo; RD: ramo direito; FAE: fascículo anterior esquerdo; FPE: fascículo posterior esquerdo; LSF: fascículo septal esquerdo; LSFB: bloqueio fascicular do septo esquerdo.
  **Figura 4:**
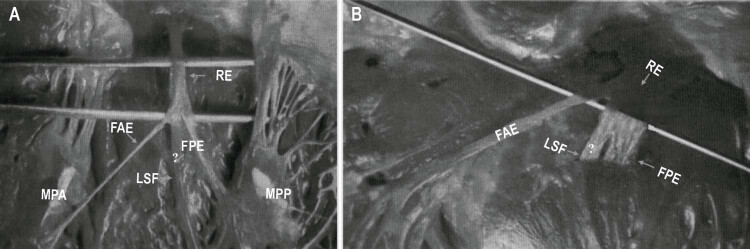
Vista lateral endocárdica do SIV no coração humano.11 Neste exemplo, o LSF se origina do ramo esquerdo (RE) principal. Além disso, o FAE conduz ao MPA da válvula mitral e o FPE diretamente ao MPP da válvula mitral (A). Figura extraída do livro original de Rosenbaum,11 o LSF se origina do FPE. Rosenbaum considerou esses como “falsos tendões” originando-se do FPE (B). RE: ramo esquerdo; RD: ramo direito; FAE: fascículo anterior esquerdo; FPE: fascículo posterior esquerdo; LSF: fascículo septal esquerdo; LSFB: bloqueio fascicular do septo esquerdo.

### Suprimento de sangue dos fascículos esquerdos

- FAE: o suprimento sanguíneo para o FAE do ramo esquerdo (RE) teve origem em 50% dos casos não apenas no ramo septal anterior da DAE, mas também da artéria nodal atrioventricular (AV), um ramo da artéria coronária direita (ACD) em 90% dos casos, e na CXE em 10%.^12^ Assim, dados anatômicos confirmam a observação de que a oclusão do segmento proximal da DAE não é um pré-requisito para a ocorrência de bloqueio fascicular anterior esquerdo (BFAE). O aparecimento de BFAE durante o infarto agudo do miocárdio não é sinal de coexistência de estenose significativa da DAE ou de doença arterial coronariana mais grave ou extensa. Nesses pacientes, outros mecanismos, como o grau da circulação colateral coronariana, podem ter um papel importante na ocorrência desse distúrbio de condução e corroboram os relatos experimentais e clínicos de que o BFAE pode estar relacionado a lesões afetando o feixe de His por meio de uma dissociação longitudinal dessa estrutura.^13^
- FPE: a ampla natureza do FPE, sua localização protegida na via de entrada do ventrículo esquerdo e seu suprimento duplo de sangue^14^ tornam o bloqueio fascicular posterior esquerdo (BFPE) isolado muito raro.^15^ O MPP onde o FPE termina é suprido por ramos arteriais que terminam na superfície diafragmática do VE e, mais comumente, por uma junção de ramos terminais da LCX e da ACD. Quando a LCX supre quase toda a superfície diafragmática do VE (10% dos corações humanos), seus ramos fornecem todo o suprimento sanguíneo para o MPP. O FPE é irrigado em 10% dos casos apenas pela DAE, em 40% dos casos pela DAE e ACD e em 50% dos casos apenas pela ACD.
- Fascículo septal esquerdo (FSE): é irrigado exclusivamente pela artéria perfurante septal da DAE, que supre 2/3 da porção superior do septo interventricular (SIV) neste local. A maior parte do suprimento de sangue para o SIV é fornecida pela DAE. Os ramos no septo da artéria descendente posterior raramente penetram mais de 10 mm do epicárdio (um pouco mais que a espessura normal da parede livre do VE), de modo que, para fins práticos, é possível considerar todo o suprimento sanguíneo do SIV derivado de quatro a seis ramos perfurantes septais de tamanho quase igual a da DAE ( Tabela 1 ).
  **Tabela 1:** Artéria responsável pela irrigação dos três fascículos do RE

Sistema responsável	FAE	FPE	LSF
Somente DAE	40%	10%	100%
DAE & ACD	50%	40%	0%
Somente ACD	10%	50%	0%

No recente artigo de consenso brasileiro, foram estabelecidos os seguintes critérios para LSFB. Eles são os seguintes, com modificações e comentários esclarecedores do nosso grupo:

- Presença de forças anteriores proeminentes (FAP) do QRS, sendo transitórias em traçados sequenciais. A natureza transitória das FAPs e as derivações envolvidas indicam uma alta probabilidade de obstrução proximal crítica da artéria coronária descendente anterior esquerda (DAE). Quando esse padrão é observado no cenário da síndrome coronariana aguda ou durante um teste de esforço, deve ser considerada uma angiografia coronariana urgente;
- Duração normal do QRS ou aumento discreto (até 110 ms) quando não associado a outros bloqueios;
- Linhas do plano frontal inalteradas;
- Tempo de pico da onda R em V1 e V2 ≥40 ms.^16^ (Nota: o termo deflexão intrínseca não é recomendado,^17^
- voltagem de onda R em V1 ≥5 mm;
- razão R/S em V1 e V2 >2;
- profundidade da onda S em V2 <5 mm;
- Possível onda q dependente da frequência cardíaca, embrionária e/ou transitória^18^ em V2 ou V1 e V2;
- Voltagem de onda R em V2> 15 mm;
- Padrões RS ou Rs em V2 e V3 (frequentemente, rS em V1) com a onda R crescente de V1 a V3 e diminuindo de V5 para V6;
- Ausência de onda q em V5, V6 e I (pela ausência do vetor septal 1_AM_);^18^ confirmado experimentalmente em corações humanos explantados por Durrer et al.,^19^

## Conclusão

Que seja de nosso conhecimento, este é o primeiro caso na literatura que descreve características de ECG compatíveis com LSFB associadas à suboclusão de TCE. Essa evolução deve alertar os clínicos sobre a possibilidade de doença arterial coronariana grave em pacientes com um padrão de ECG de LSFB associado ao infradesnivelamento generalizado do segmento ST, tanto em pacientes com angina estável quanto naqueles com síndrome coronariana aguda. Deve-se considerar a angiografia coronariana o mais rapidamente possível.
