# Supplementary material for: Forças Anteriores Proeminentes do QRS Durante Suboclusão Transitória do Tronco da Coronária Esquerda
Source: Arq Bras Cardiol. 2020 Sep 11;115(1 Suppl 1):1–5. [Article in Portuguese] doi: 10.36660/abc.20180363 (PMC8386954; doi:10.36660/abc.20180363)
Supplement: Translation

# Translation: Transient Prominent Anterior QRS Forces in Acute Left Main Coronary Artery Subocclusion: Transient Left Septal Fascicular Block

**Authors:** Andrés Ricardo Pérez-Riera, Raimundo Barbosa-Barros, Rodrigo Daminello Raimundo, Luiz Carlos de Abreu, Marcos Célio de Almeida, Kjell Nikus

**Affiliations:** 1 Brazil Centro Universitario Saúde ABC, Santo André, SP - Brazil; 2 Brazil Hospital de Messejana Dr. Carlos Alberto Studart Gomes, Fortaleza, CE - Brazil; 3 Brazil Universidade de Brasília - Instituto de Biologia-Genética e Morfologia, Brasilia, DF - Brazil; 4 Finland Heart Center, Tampere University Hospital and Faculty of Medicine and Life Sciences, Tampere – Finland

**Keywords:** Coronary Occlusion, Truncus Arterious, Acute Coronary Syndrome, Fibrinolytic Agents, Percutaneous Coronary Intervention, Angina, Stable, Electrocardiography/methods

## Introduction

The left main coronary artery (LMCA) originates from the left sinus of Valsalva, passes between the main pulmonary artery and the left atrial appendage before entering the coronary sulcus and bifurcates into the left anterior descending (LAD) and the left circumflex (LCX) coronary arteries. In most individuals, the LMCA supplies ≈75% of the left ventricle (LV).^1^ Significant stenosis, which may lead to stable angina and/or acute coronary syndrome, places the patient at risk of life-threatening acute left ventricular failure and malignant arrhythmias. Patient prognosis of LMCA disease can be improved with coronary artery bypass grafting (CABG). With technical improvement and effective anti-thrombotic medication, percutaneous coronary intervention (PCI) has evolved as an alternative therapeutic modality. In patients with severe LMCA disease having low to intermediate anatomic complexity, both CABG and PCI are effective methods of revascularization with comparable long-term rates of death, myocardial infarction, and stroke.^2^ Patients most suitable for LMCA stenting are those with isolated ostial/mid LMCA disease, protected LMCA disease and those who undergo an elective stenting procedure. In a recent study, 8% mortality and 8% target lesion revascularization rate during one-year follow-up was reported.^3^

## Case description

A 72-year-old Caucasian male presented at the emergency department complaining of prolonged oppressive chest pain at rest since ≈1 hour associated with cold diaphoresis and respiratory distress. He had a history of type 2 diabetes mellitus and dyslipidemia had been detected four years before. Two months earlier, he had oppressive precordial pain on moderate exertion, which disappeared rapidly after rest. Figure 1 shows the ECG at admission and Figure 2-A an ECG performed 30 days before. The coronary angiography indicated subocclusion (91-99% diameter stenosis) in the middle portion of the LMCA ( Figure 2-B ). CABG was immediately proposed, but the patient refused. He successfully underwent PCI with DES implantation without in-hospital complications. During the 6-month follow-up no target lesion revascularization was required on the LMCA. The patient remained asymptomatic even at efforts and several follow-up ECGs were normal.

**Figure 1:** – ECG at admission (A) and the injury vector in the frontal and horizontal planes (B). A) Widespread ST-segment depression in I, II, III (II>III) and VF and from V2 to V6 waves. Diffuse ST-segment depression in the inferolateral leads (≥7 leads with ST-depression) and reciprocal ST-segment elevation in the aVR lead. In addition, atypical left anterior fascicular block (LAFB), QRS axis -40°, SIII>SII, and absence of initial q wave in I and aVL by absence of the first left middle septal vector (in typical LAFB the first 10-20 ms vectors are directed to +120°).4 B) Frontal plane (FP): The ST injury vector (arrow) is directed upward and rightward pointing towards the aVR lead (-150°). When this vector is located between -90° and ±180° in the FP, it is indicative of LMCA obstruction in up to 100% of cases;5 ST-segment depression in the inferior leads with STII>STIII; Horizontal plane: the ST injury vector is directed to the right and leftward (arrow), perpendicular to V1. ST segment depression from V2 to V6.

**Figure 2:** – A) ECG performed 30 days before: left atrial enlargement, prominent anterior QRS forces in V2 with qRs pattern in V1-V2, R wave voltage in V2 >15 mm (23 mm), prolonged R-wave peak time in right precordial leads (≥35 ms), ST-segment elevation in aVR (≥1 mm), minimal ST-segment depression in the inferior leads and from V3 to V6; these discrete alterations could raise the suspicion of LMCA disease and some degree of LSFB. Note: this ECG was considered “normal” by the clinician!!B) Coronary angiography in right anterior oblique cranial projection: this view shows a critical sub-occlusion of the LMCA (arrow) in the middle portion.

## Discussion

An ECG performed due to stable angina symptoms 30 days before the hospital admission, showed a pattern suggestive of LMCA disease and possibly some degree of LSFB.^4 , 5^ These “minimal findings” in the scenario of stable angina should alert the clinician about the possibility of severe myocardial ischemia in patients without a logical explanation for the ECG findings, such as left ventricular hypertrophy with strain in structural heart disease. Both ECG features are evident, with more pronounced ischemic findings in the ECG performed at admission when the patient had acute coronary syndrome.

Several successive manuscripts from our group and others have shown that a large proportion of cases with transient left septal fascicular block (LSFB), manifested by prominent anterior QRS forces, is caused by critical proximal obstruction of the LAD before its first septal perforator branch.^6 - 9^ As the LAD is a continuation of the LMCA, significant LMCA obstruction, may lead to ischemia in the mid-portion and apical territory of the left ventricle, where the left septal fascicle runs, thereby causing LSFB.

In the presence of LSFB, the sequence of ventricular activation begins only at two points:

The base of the anterolateral papillary muscle (ALPM) of the mitral valve dependent on the left anterior fascicle (LAF) in the anterior paraseptal wall, just below the ALPM attachment (1_AM_vector);

The base of the posteromedial papillary muscle of the mitral valve (PMPM) dependent on the left posterior fascicle (LPF). It is located in the posterior paraseptal wall, at about one-third of the distance from the apex to the base (posteroinferior vector – 1_PI_). These two initial vectors have opposite directions, and they cancel each other with minimal predominance of the 1_PI_vector directed backward ( Figure 3 ). This explains the absence of the normal initial convexity to the right of the QRS loop in the horizontal plane, dependent on the 1_AM_septal vector (or Penaloza-Tranchesi vector).^10^ Next, the stimulus is directed to the mid-septal or left paraseptal region, blocked by numerous Purkinje passage areas, thus shifting the forces to the front and the left, resulting in prominent anterior forces (PAF). Figure 4 shows two cases where the trifascicular anatomy of the left His system is evident. Ironically, both cases come from the school of electrocardiography that coined the bifascicular concept of the left His system.^11^

**Figure 3:** – Outline showing the initial ventricular activation in cases of LSFB. Left His system with its three divisions, in a left sagittal projection. The LAF ends at the base of the ALPM of the mitral valve. The LPF ends in the base of the PMPM of the mitral valve. Since the activation vectors dependent on the anterosuperior (A) and posteroinferior (B) fascicles go in opposite directions, they cancel each other, with minimal predominance of LPF. This phenomenon explains the frequent initial q wave in the right precordial leads in the presence of LPFB. Note the absence of the first 1AM vector, dependent on LSF. LBB: left bundle branch; RBB: right bundle branch; LAF: left anterior fascicle; LPF: left posterior fascicle; LSF: left septal fascicle; LSFB: left septal fascicular block.

**Figure 4:** – Endocardial lateral view of the IVS in the human heart.11 In this example the LSF originates from the main LBB. Additionally, the LAF conducts to the ALPM of the mitral valve and the LPF straight to the PMPM of the mitral valve (A). Figure extracted from the original book by Rosenbaum,11 the LSF originates from the LPF. Rosenbaum considered these as “false sinews or tendons” originating from the LPF (B). LBB: left bundle branch; RBB: right bundle branch; LAF: left anterior fascicle; LPF: left posterior fascicle; LSF: left septal fascicle; LSFB: left septal fascicular block.

### Left fascicles blood supply

**LAF:** the blood supply to the LAF of the LBB originated in 50 % of the cases not only from the anterior septal branch of the LAD, but also from the atrioventricular (AV) nodal artery, a branch of the right coronary artery (RCA) in 90 % of the cases and of the LCX in 10%.^12^ Thus, anatomic data support the observation that occlusion of the proximal segment of the LAD is not a prerequisite for the occurrence of LAFB. The appearance of LAFB during acute myocardial infarction is not a sign of a coexistent significant stenosis of the LAD or of more severe or extensive coronary artery disease. In these patients, other mechanisms such as the degree of the coronary collateral circulation may play a role in the occurrence of this conduction disturbance and supports the experimental and clinical reports that LAFB may be due to lesions involving the His bundle by means of a longitudinal dissociation of this structure.^13^

**LPF:** the broad nature of the LPF, its protected location in the left ventricular inflow tract, as well as its dual blood supply^14^ makes isolated LPFB very rare.^15^ The PMPM where LPF ends is supplied by arterial branches that terminate on the diaphragmatic surface of the LV, and most commonly by a junction of terminal branches of the LCX and of the RCA. When the LCX supplies nearly all the diaphragmatic surface of the LV (10 % of human hearts), its branches provide the entire blood supply for the PMPM. The LPF is irrigated in 10% of cases by LAD only, in 40% of cases by LAD and RCA and in 50% of cases by RCA only.

**Left septal fascicle (LSF):** it is irrigated exclusively by the septal perforating artery from the LAD, which supplies the upper^2^ /_3_portion of the interventricular septum (IVS) at this site. Most of the blood supply to the IVS is provided by the LAD. Branches into the septum from the posterior descending artery rarely penetrate more than 10 mm from the epicardium (slightly more than the normal thickness of the LV free wall), so that for practical purposes one may consider the entire blood supply of the IVS to be derived from four to six nearly equal size septal perforating branches of the LAD ( Table 1 ).

**Table 1:** – Artery responsible for the irrigation of the three fascicles of the LBB

Responsible system	LAF	LPF	LSF
LAD only	40%	10%	100%
LAD & RCA	50%	40%	0%
RCA only	10%	50%	0%

In the recent Brazilian consensus paper, the following criteria for LSFB were established. They are as follows, with modifications and clarifying comments by our group:

Presence of prominent anterior forces (PAFs) of the QRS, being transient in sequential tracings. The transitory nature of PAF and the leads involved in it indicate a high likelihood of critical proximal obstruction of the left anterior descending coronary artery (LAD). When this pattern is observed in the scenario of acute coronary syndrome or during a stress test, urgent coronary angiography should be considered;

Normal QRS duration or discrete increase (up to 110 ms) when not associated to other blocks;

Unaltered frontal plane leads;

R-wave-peak time in V1 and V2 ≥40 ms.^16^ (Note: the term intrinsicoid deflection is not recommended,^17^

R-wave voltage in V1 ≥5 mm;

R/S ratio in V1 and V2 >2;

S-wave depth in V2 <5 mm;

Possible heart-rate dependent, embryonic and/or transient q wave^18^ in V2 or V1 and V2;

R-wave voltage in V2 >15 mm;

RS or Rs patterns in V2 and V3 (frequently, rS in V1) with R wave “in crescendo” from V1 through V3 and decreasing from V5 to V6;

Absence of q wave in V5, V6 and I (by absence of 1_AM_septal vector)^18^ ; confirmed experimentally in explanted human hearts by Durrer et al.^19^

## Conclusion

To our knowledge, this is the first case in the literature describing ECG features compatible with LSFB associated with LMCA subocclusion. This evolution should alert clinicians about the possibility of severe coronary artery disease in patients with an ECG pattern of LSFB associated with wide-spread ST-segment depression both in patients with stable angina and those with acute coronary syndrome. Coronary angiography without delay should be considered.
